# Necrosis of the Superior Maxillary

**Published:** 1897-11

**Authors:** Olin M. Drake

**Affiliations:** Boston


### NECROSIS OF THE SUPERIOR MAXILLARY.

Olin M. Drake, M. D., Boston.

Mr. S. consulted me January 13th, 1896, for the following: Some six or seven weeks previously there appeared upon the upper left jaw what he supposed was a gum boil, but soon extended considerably along the gum anteriorly. This swelling eventually broke, discharging a thick, yellow, offensive pus. His dentist pronounced his trouble necrosis with fistula. He suggested that the canine and the first and second bicuspids should be extracted, and the alveolar processes and jaw cleared of all dead bone. The patient would not submit to this operation, and therefore consulted me. I found the jaw very much swollen from the left central incisor to the wisdom tooth; just back of the eye-tooth was an opening from which could be easily pressed a light yellowish, offensive pus. There was but little pain, only a sensitiveness or soreness of the teeth involved. They felt much elongated when closing the teeth. The left central incisor was the most troublesome of them all. Otherwise the patient did not suffer. I first prescribed Fluoric-acid 200th, but as no improvement followed its administration, on the 23d I gave him Silicea 200th. Sixteen days later the swelling had diminished and the teeth were less sensitive, and small pieces of bone were being discharged every day or so.

I then gave him Silicea 10m, which, I suppose, many of you would think wrong. At the expiration of ten days (February 29th) I found the swelling and sensitiveness gone, and a piece of bone was shown me, very thin and as long as my little finger nail, but not as wide, which had come away, besides smaller pieces. There was still a discharge of thin, offensive pus. I prescribed Silicea 72m. On March 11th there was a report of further continued improvement, and March 20th he was still gaining. Saccharum-lactis was given on both of the above occasions. I did not see patient again until May 6th, when all swelling and sensitiveness were gone. The fistula was still discharging, but scantily and without fetor. I now gave Hekla-lava 30th, and two weeks later there was nothing to be noticed or complained of, objectively or subjectively, in my patient.

